# Detection of multidrug-resistant organisms of concern including *Stenotrophomonas maltophilia* and *Burkholderia cepacia* at a referral hospital in Kenya

**DOI:** 10.1371/journal.pone.0298873

**Published:** 2024-04-16

**Authors:** Racheal Kimani, Patrick Wakaba, Moses Kamita, David Mbogo, Winnie Mutai, Charchil Ayodo, Essuman Suliman, Bernard N. Kanoi, Jesse Gitaka

**Affiliations:** 1 Centre for Research in Infectious Diseases, Directorate of Research Innovation, Mount Kenya University, Thika, Kenya; 2 Thika Level V Hospital, Thika, Kenya; 3 Department of Medical Microbiology & Immunology, University of Nairobi, Nairobi, Kenya; 4 Washington State University Global Health-Kenya, Nairobi, Kenya; 5 Department of Microbiology, Mount Kenya University, Thika, Kenya; Hawassa University College of Medicine and Health Sciences, ETHIOPIA

## Abstract

Regular monitoring of bacterial susceptibility to antibiotics in clinical settings is key for ascertaining the current trends as well as re-establish empirical therapy. This study aimed to determine bacterial contaminants and their antimicrobial susceptibility patterns from medical equipment, inanimate surfaces and clinical samples obtained from Thika Level V Hospital (TLVH), Thika, in Central Kenya. Three hundred and five samples were collected between the period of March 2021 to November 2021 and comprised urine, pus swabs, catheter swabs, stool, and environmental samples. Bacterial identification and antimicrobial susceptibility were performed using VITEK 2 and disc diffusion respectively. We observed that Coagulase-negative *Staphylococci* (28 /160, 17.5%) were the most commonly isolated species from clinical samples followed by *E*. *coli* (22 /160 13.8%) and *S*. *aureus* (22/160, 13.8%). The bed rails were the mostly contaminated surface with *S*. *aureus* accounting for 14.2% (6/42). Among the clinical samples, pus swabs yielded the highest number of pathogens was pus (92/160). Trauma patients had the highest proportion of isolates (67/160, 41.8%). High level of antimicrobial resistance to key antimicrobials, particularly among Enterobacterales was observed. Extended Spectrum Beta Lactamase (ESBL) phenotype was noted in 65.9% (29/44) of enteric isolates. While further ESBL genetic confirmatory studies are needed, this study highlights the urgent need for actions that mitigate the spread of antibiotic-resistant bacteria.

## Introduction

The emergence of multi-drug resistant (MDR) strains in healthcare facilities poses a threat to the management of infections in developing countries [1]. Approximately 700,000 people die annually from antimicrobial-resistant (AMR) infections globally [2], with a projected 10 million deaths by 2050 [3]. In Kenya, high levels of hospital-acquired infections (HAIs) have been reported signifying the need for reinforced infection prevention control strategies [4–6]. Multidrug-resistant bacterial contaminants have been reported in hospital wards in Kenya [7] albeit in a small number of hospitals.

At present, β-lactam drugs are a key factor in the treatment of bacterial infections worldwide and account for almost 65% of antibiotic usage [1]. These drugs have been classified into six main groups based on the chemical structure of the β-lactam ring, including penicillins, cephalosporins, cephamycins, carbapenems, monobactams, and β-lactamase inhibitors. Unfortunately, despite their crucial role in combating infections, recent years have witnessed a concerning global rise in resistance to this important class of antibiotics [8].

Both community-acquired infections (CAIs) and HAIs causative agents have seen a considerable increase in antimicrobial resistance [9]. The hospital environment, in particular, serves as a source of multidrug-resistant organisms (MDROs), especially when routine practices such as environmental disinfection, contact precautions, and hand hygiene are not consistently implemented [10]. Pathogens reported to persist in these healthcare environments include *Clostridium difficile* spores, vancomycin-resistant Enterococcus (VRE), methicillin-resistant *Staphylococcus aureus* (MRSA), and *Acinetobacter baumannii* [11]. High-risk areas for contamination include hard surfaces like beds, handles, and grab bars. Transmission of pathogens to patients is thought to be through healthcare workers, visitors, or asymptomatic carriers [12]. Although enhanced cleaning of hospital surfaces has been advocated to overcome the transmission of MDROs, a study conducted in 2008 examined the cleaning of hospital surfaces in 36 acute care hospitals in the USA and found that only 47% of these surfaces were effectively cleaned [13]. This represents a critical area that needs attention in the fight against the spread of MDROs.

In case of CAI, resistance can be attributable to the widespread use of broad-spectrum antibiotics, over the counter sale of antibiotics, self-administration of antibiotics, inappropriate use of antibiotics, and a lack of compliance with treatment [14, 15]. Conversely, in the case of HAIs, causative agent antimicrobial resistance is mainly due to the extensive use of broad-spectrum antibiotics in their management. Among the microorganisms usually implicated in HAIs include *Pseudomonas aeruginosa*, *Escherichia coli*, *Staphylococcus aureus*, *Klebsiella species*, and coagulase-negative *Staphylococci*. Some of the factors that contribute to the development of HAIs includes hospital design factors (e.g., ventilation and an adequate number of handwash basins) [16], longer hospital stays, gender, surgery since admission, intubation, mechanical ventilation, age of the patient, type of hospital, and urinary catheter and hygienic practices [17].

In Kenya, the past decade has seen a significant increase in efforts to describe and address the burden of drug-resistant infections in the country. These efforts have increasingly focused on hospitals like the Thika Level V Hospital in Thika. By participating in these initiatives, Kenya aims to eventually contribute AMR related data to global platforms such as the World Health Organization’s Global Antimicrobial Resistance Surveillance System (GLASS) [18]. Thus, this study aimed to determine bacterial contaminants and their antimicrobial susceptibility patterns from medical equipment, and inanimate surfaces at the Thika Level V Hospital. The study further determined the antibiotic resistance profile for each clinically important isolate. We observed high levels of resistance to key antimicrobials, especially by enteric bacteria.

## Materials and methods

### Sampling

A convenience sampling technique was used to enrol cases. The following formula: z2 × p (1‐p)/e2 [z  =  z‐score  =  1.96, e  =  margin of error  =  5%, and p  =  standard of deviation] was used to estimate the optimum sample size with confidence interval level of 95%. Based on the previous study by Greenhow *et al*., [19] the bacterial infections prevalence was recognized in 3.75% of clinical samples (95% confidence interval: 0.4%–6.7%). Hence, we select the p  =  standard of deviation as 5.0% and accordingly the estimated sample size was calculated as 56 patients. However, we surpassed this number and collected urine, pus and stool samples from 90 patients. The samples from the environment were representative samples from each of the selected departments.

### Study population and sample collection

A total of 305 samples were collected at Thika Level V Hospital. 200 were clinical samples and 105 were samples from high contact surfaces. Thika Level 5 Hospital is a 300-bed Government Hospital in the town of Thika, approximately 50 km northeast of Nairobi, in Central Province, Kenya. It provides medical, surgical, gynaecological, obstetric, and paediatric services to a mixed urban and rural population and has a total of seven inpatient wards. Patient sociodemographic, and clinical data (diagnosis and treatment received) were filled in pathological data forms. Urine, pus swabs, stool, and indwelling catheter swabs were aseptically collected from patients. The age bracket of our participants was 17 years to 96 years. Environmental swab samples from high-contact surfaces including the bed rail, intravenous fluid pole, cabinets, medicine tray, monitors, nurses’ working stations, doorknobs, and sphygmomanometer were also collected. Sampling was done by use of a moist sterile swab passed over the surfaces of inanimate objects and equipment. The patients sampled included those from the renal unit, the ICU, and the male medical and surgical wards (male and female). A proportion of the renal unit patients were not admitted to the hospital. However, we didn’t establish whether the patients had community-acquired infections at the time of admission for surgery. A small proportion of patients admitted in the medical ward was primarily hospitalized due to infections (those with cellulitis, gangrene, and diabetic foot).

#### Sample processing

The samples were transported under chilled conditions to the Mount Kenya University, Centre for Research, and Infectious Diseases Laboratory, for processing where they were cultured on selected media plates. In brief, to isolate bacteria from the hospital, samples collected included pus swabs and highly touched surface swabs and were inoculated on Blood agar, MacConkey agar, and Mannitol Salt agar media. Surface swabs had been immersed in sterile saline before swabbing the points of interest. Urine samples were inoculated on Blood Agar, MacConkey agar and CLED (Cysteine Lactose Electrolyte Deficient) agar. Stool samples were inoculated on MacConkey Agar alone. All media were incubated at 37°C for 18hrs. Based on colonial morphology on different culture plates, two or more colonies were further characterized by first subculturing on specific media, followed by gram staining. The isolates were then identified by VITEK 2 using Gram negative (GN) and Gram-positive (GP) identification cards (ID). For identification, one colony was picked aseptically from each pure colony and emulsified on a sterile normal saline solution using a sterile cotton swab. A homogenous organism suspension formed was adjusted to the required McFarland standard (0.5–0.63). Turbidity of 0.5–0.6 is recommended for inoculum preparation in VITEK (bioMérieux). The McFarland turbidity was checked by an instrument, DensiCHEK Plus, as per the manufacturer’s (bioMérieux) guidelines. This was introduced to the machine probe which suctioned the suspension and introduced to the card. After four hours the results were obtained from the computer giving the identity of the bacteria.

#### Antimicrobial susceptibility testing for Gram-negative bacteria

Antimicrobial susceptibility tests were performed by the standard Kirby–Bauer disk diffusion method on the Mueller–Hinton agar media (Thermo Scientific™ Oxoid™) using commercially available antibiotic disks (Oxoid™, UK). The diameter of the inhibition zone was measured for each antibiotic disk, and the results were defined as per the CLSI guidelines [20]. The antibiotics used against Gram-negative isolates included cefuroxime (30μg), ceftriaxone (30μg), cefotaxime (30μg), piperacillin (30μg), sulphamethoxazole (25μg), amoxicillin-clavulanate (10μg), gentamycin (10μg), aztreonam (30μg), ceftazidime (30μg), ciprofloxacin (5μg), cefoxitin (30μg), cefepime (30μg), minocycline (30μg), levofloxacin (5μg), amikacin (30μg), piptazobactam (110μg), imipenem (10μg), meropenem (10μg), tetracycline (30μg), nalidixic acid (10μg) and ampicillin (10μg). For *Staphylococci* species antibiotics tested included erythromycin (15 μg), amoxycillin-clavulanate (30 μg), sulphamethoxazole/trimethoprim (25 μg), penicillin (10IU), tetracycline (30 μg), gentamycin (10 μg), clindamycin (2 μg), oxacillin (1 μg), cloxacillin (5 μg), levofloxacin (5 μg), cefoxitin (30 μg), and ceftriaxone (30 μg). Gram-negative isolates that were resistant to cefotaxime, ceftriaxone, ceftazidime, and aztreonam were presumed to be ESBL producers whereas *Staphylococci* isolates resistant to cefoxitin, cloxacillin and oxacillin were presumed to be methicillin resistant. AST breakpoints were interpreted according to CLSI M100 guidelines [20].

### Quality control

Quality control measures on media for culture were run before carrying out actual cultures to confirm the sterility and selectivity of media used. Quality control organisms during VITEK identification included *Staphylococcus saprophyticus* (ATCC BAA-750) for the Gram-positive ID cards and *Enterobacter cloacae* (ATCC 700323) for the Gram-negative cards. For antimicrobial susceptibility testing, quality control testing was achieved using ATCC microorganisms *S*. *aureus* 29213 *E*. *coli* 25922, and *K*. *pneumoniae* 700603.

### Ethical considerations

This study was approved by the Research and Ethics Committee of Mount Kenya University (MKU/ERC/1687) dated 18^th^ November 2020 and licensed by the National Commission for Science, Technology, and Innovation (NACOSTI) (NACOSTI /P/21/7678). Before enrolment, informed consent was obtained from all the study participants or their legal guardians for participants under the age of 18 years. Anonymized patients’ demographic characteristics were recorded on the pathological investigation forms, which were securely filed and kept under lock and key, for confidentiality.

### Data analysis

The socio-demographic, clinical diagnosis, treatment received, and microbiological data were collectively documented for each patient on a Microsoft Excel sheet. The environmental data were also documented accordingly. Kruskal–Wallis nonparametric test was used to test the differences in resistance between species using the SPSS statistical package version 26. Discrete data was described using means, while the categorical data were computed as frequencies, with the data presented in tables and figures.

## Results

### Patients baseline information

The mean age of participants was 46 years, and the range was 17 years to 96 years. The majority of positive cultures were from the age group 27–36 (39/160; 24.3%) and 37–46 (34/160, 21.2%) years old as seen in **Table 1**. Patient presented with a range of conditions including decubitus ulcers, burns, peritonitis, trauma, septic wounds, gangrene, diabetic foot, end-stage renal disease, cellulitis, and congestive cardiac failure. The main antibiotics administered to patients were ceftriaxone, metronidazole, and flucloxacillin.

**Table 1:** Demographic information of the participants and distribution of bacterial species by age group.

Result by age group	Sex		17–26	27–36	37–46	47–56	57–66	67–76	77–86	87–96	Total
	Male	Female									
No bacteria growth	43	100									143
Positive culture	119	41									160
***Enterobacteriaceae***											
*Escherichia coli*	13	9	0	3	4	1	7	4	3	1	22
*Klebsiella pneumoniae*	4	2	3	1	1	0	1	0	0	0	6
*Klebsiella oxytoca*	1	0	0	1	0	0	0	0	0	0	1
*Proteus mirabilis*	2	1	1	0	0	1	0	0	2	0	4
*Salmonella enterica*	0	1	0	0	0	0	0	0	0	1	1
*Enterobacter cloacae*	9	1	0	2	6	0	1	0	1	0	10
***Streptococacae***											
*Enterococcus faecalis*	4	4	1	0	3	0	1	3	0	0	8
*Staphylococcacae*											
*Cn Staphylococci*	15	13	10	7	3	2	2	1	1	2	28
*Staphylococcus aureus*	21	1	6	7	3	2	5	2	0	0	22
***Pseudomonacae***											
*Pseudomonas aeruginosa*	1	0	1	0	0	0	0	0	0	0	1
*Pseudomonas luteola*	7	1	1	3	0	3	0	1	0	0	8
*Pseudomonas oryzihabitans*	1	0	0	0	0	1	0	0	0	0	1
*Acinetobacter baumannii*	17	2	1	8	5	1	1	0	0	3	19
*Acinetobacter lowflii*	1	0	0	1	0	0	0	0	0	0	1
*Others*	20	7	3	5	8	6	0	0	3	2	27

### Bacterial species identified

**Fig 1:**
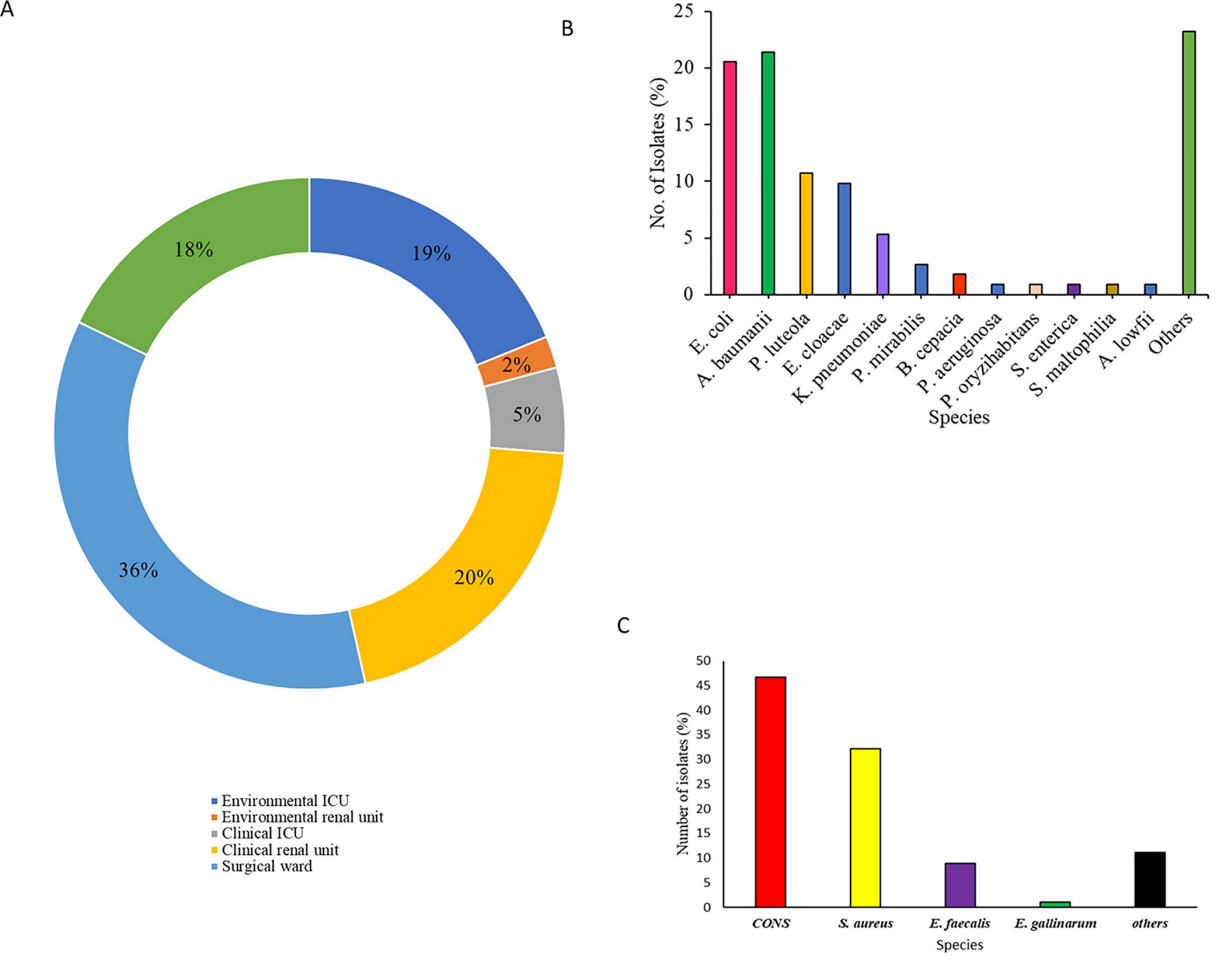
a) General distribution of bacterial isolates from patients and inanimate objects b) Spectrum of isolated gram-negative species; c) Isolated gram-positive species.

Out of 305 culture-positive plates (excluding mixed growth and fungal growth), 202 (66.2%) were culture-positive; 160 from 90 clinicals samples and 42 from 44 hospital surfaces and equipment swabs. The highest prevalence of isolates was reported in the surgical ward with 72/202 (35.6%) isolates, followed by the intensive care unit with 49/202 (24.2%), renal unit with 45/202 (22.2%) isolates, and the medical ward with 36/202 (17.8%) isolates **(Fig 1A).** Gram-negative isolates (55.4%, 112/202) were more than the Gram-positive isolates (90/202, 44.6%)**. Fig 1B and 1C** show all isolates identified from the samples analysed.Gram-positive isolates included CONS, *S*. *aureus*, and *Enterococci* spp amongst others. Gram-negative species were mainly enteric bacteria, *Acinetobacter* spp, and *Pseudomonas* spp, amongst others.

### Bacterial isolates from patients by the sample types and hospital wards

**Fig 2:**
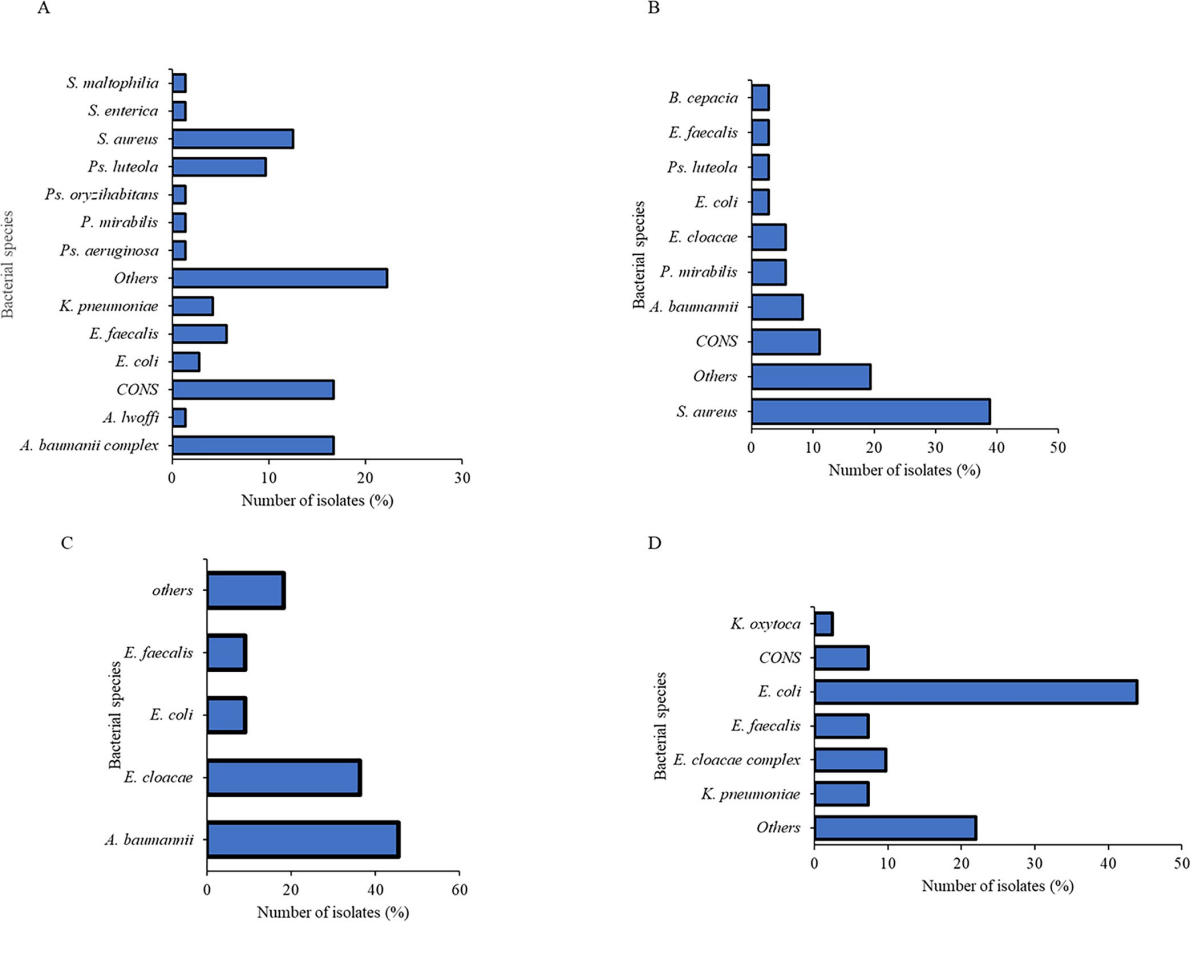
Isolates obtained from patients (A) shows isolates from surgical ward patients; (B) shows isolates from medical ward patients isolates; (C) shows isolates obtained from ICU patients and (D) shows isolates from renal unit patients.

Coagulase-negative *Staphylococci* (28/160, 17.5%) were the most isolated species followed by *S*. *aureus* (25/160, 15.6%) and *E*. *coli* (22/160, 13.8%) from clinical samples. The clinical sample with the highest number of pathogens was pus swabs (92/160, 57.55%), followed by urine samples (56/160, 35%), stool (11/160, 6.87%), and lastly was the exit site swab (1/160, 0.7%). *E*. *coli*, *K*. *pneumoniae*, *K*. *oxytoca*, *P*. *mirabilis*, *S*. *enterica*, *E*. *cloacae*, *A*. *baumannii*, CONS, *S*. *aureus*, and *E*. *faecalis* were all significant isolates from samples collected in this study. CONS and *S*. *aureus* are normal flora of the skin and normally contaminate wounds and the urinary tract to cause infections in their unusual sites of habitation. The rate of isolation of other significant pathogens from the samples such as *E*. *cloacae* was (10/160, 6.25%) and *E*. *faecalis* (8/160, 5%). The most dominant isolates from urine were *E*. *coli* (12/56, 21.4%) followed by *A*. *baumannii* (7/56, 12.5%). *K*. *pneumoniae* was mainly isolated from stool and pus swabs. *E*. *cloacae* and *E*. *feacalis* were mainly found in pus 4/92 4.34% and urine samples (4/56, 7.14%) (**Table 2)**. Among the isolates from surgical ward, *A*. *baumannii* accounted for the highest percentage (16.7%, 12/72) **(Fig 2A).** *S*. *aureus* (27.8%, 10/36) was predominant among all medical ward isolates **(Fig 2B).** Among the isolates from clinical samples from ICU, *A*. *baumannii* was the most common at 36.3% (5/11) of all isolates in this section (**Fig 2C)**. The clinical renal unit isolates depicted that *E*. *coli* was the highest in number among all isolates with a percentage of 43.9% (18/41) (**Fig 2D).**

**Table 2:** Distribution of identified species from different samples.

			Sample		
	Urine	Pus	Stool	Exit site swab	Total
Renal Unit	28 (70%)	3 (7.5%)	9 (22.5%)	1 (2.4%)	41 (25.6%)
ICU	9 (81.8%)	0 (0%)	2 (18.2%)	0 (0%)	11 (6.9%)
Medical Ward	4 (11.1%)	32 (88.9%)	0 (0%)	0 (0%)	36 (22.5%)
Surgical Ward	15 (20.8%)	57 (79.2%)	0 (0%)	0 (0%)	72 (45%)
Total	56 (35%)	92 (57.5%)	11 (6.9%)	1 (0.63%)	160
*Enterobacteriaceae*					
*E*. *coli*	12 (21.4%)	3 (3.3%)	7 (63.6%)	0 (0%)	22 (13.8%)
*K*. *pneumoniae*	0 (0%)	3 (3.3%)	2 (18.2%)	0 (0%)	5 (3.1%)
*K*. *oxytoca*	0 (0%)	0 (0%)	1 (9.1%)	0 (0%)	1 (0.6%)
*P*. *mirabilis*	0 (0%)	3 (3.3%)	0 (0%)	0 (0%)	3 (1.9%)
*S*. *enterica*	0 (0%)	1 (1.1%)	0 (0%)	0 (0%)	1 (0.6%)
*E*. *cloacae*	7(12.5%)	2 (2.2%)	0 (0%)	0 (0%)	9 (5.6%)
*Streptococacae*					
*E*. *faecalis*	4 (7.1%)	4 (4.3%)	0 (0%)	0 (0%)	8 (5%)
*Staphylococacae*					
*Cn Staphylococci*	7 (12.5%)	20 (21.7%)	0 (0%)	1 (100%)	28 (17.5%)
*S*. *aureus*	1 (1.8%)	24(26.1%)	0 (0%)	0 (0%)	25 (15.6%)
*Pseudomonaceae*					
*P*. *aeruginosa*	0 (0%)	1 (1.1%)	0 (0%)	0 (0%)	1 (0.6%)
*P*. *luteola*	2 (3.6%)	5 (5.4%)	0 (0%)	0 (0%)	7 (4.4%)
*P*. *oryzihabitans*	0 (0%)	1 (1.1%)	0 (0%)	0 (0%)	1 (0.6%)
*A*. *baumanii*	7 (12.5%)	13(14.1%)	0 (0%)	0 (0%)	20 (12.5%)
*A*. *lwoffii*	1 (1.8%)	0 (0%)	0 (0%)	0 (0%)	1 (0.6%)
Others	15 (26.8%)	12 (13%)	1 (9.1%)	0 (0%)	28 (17.5%)

### Distribution of bacterial isolates by patients’ conditions

**Fig 3:**
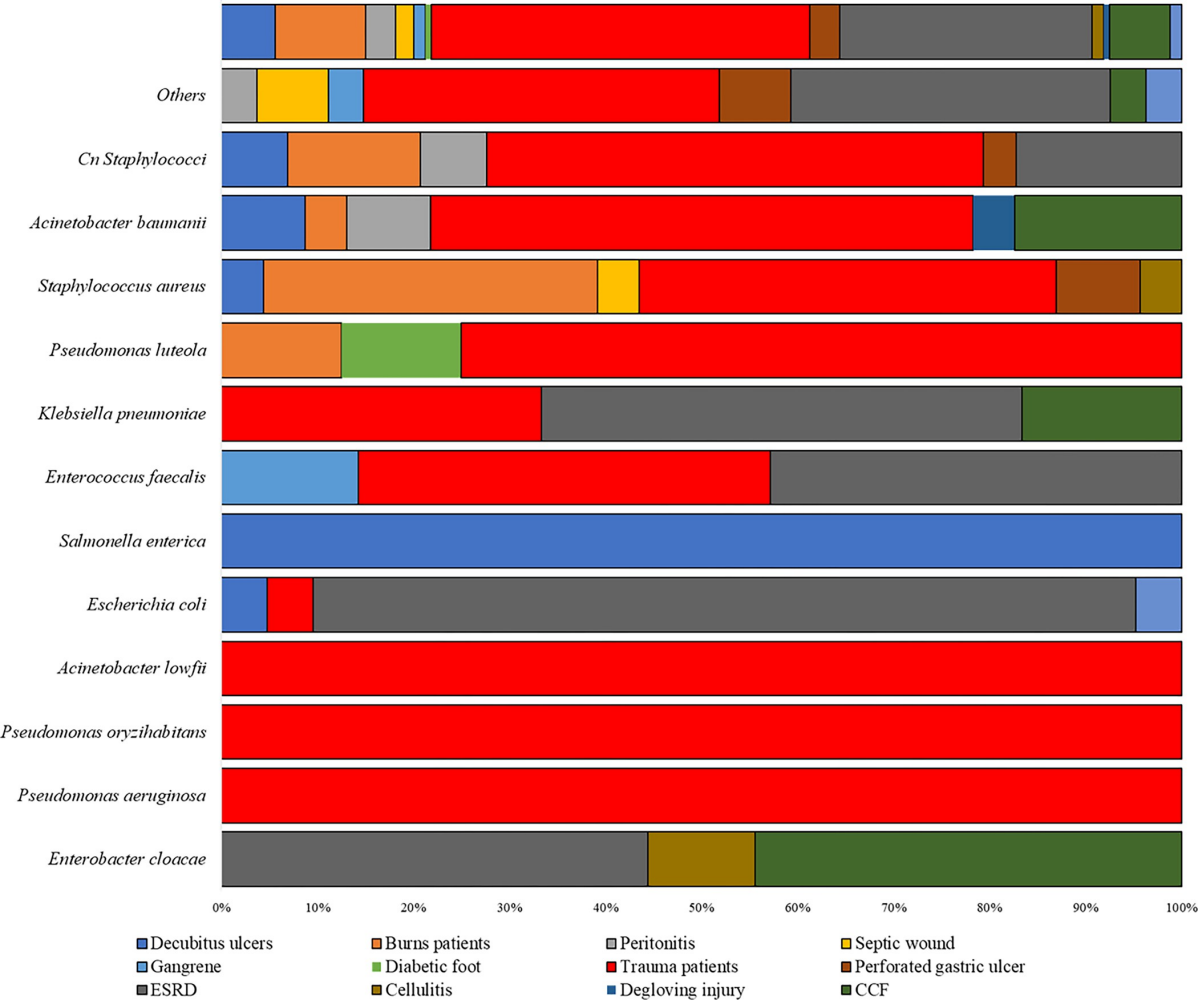
Distribution of various bacteria species isolated by patients’ presentations.

The majority of bacterial isolates were from trauma patients in the surgical ward (67/160, 41.8%). The other bacterial isolates were distributed among patients with burns 14/160 (8.75%), peritonitis patients 5/160, (3.1%), septic wound patients 3/160 (1.9%), decubitus ulcers patients 8/160 (5%) and renal unit patients with end-stage renal disease (ESRD) 42/160 (26.23%) and perforated gastric ulcer 4/160 (2.5%) among others **(Fig 3).**

### Bacterial isolates from hospital environment surfaces and inanimate objects

**Fig 4:**
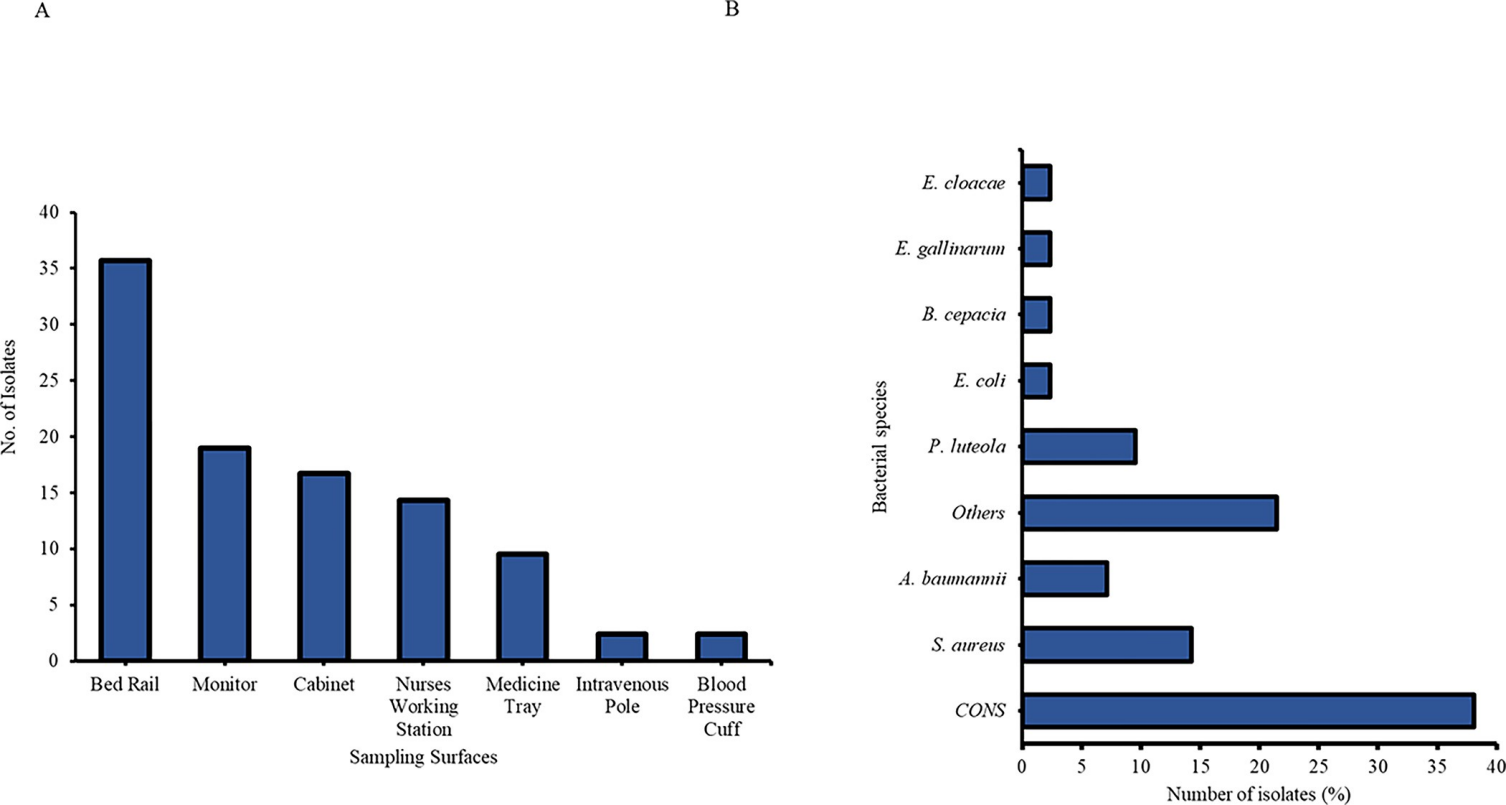
Bacterial isolates from hospital environment (A) Distribution on high touch surfaces (B) Type of isolates from high touch surfaces.

The bed rail was the most contaminated inanimate object sampled at 38% (16/42 isolates). This was followed closely by the monitors, which had 8/42 (19.0%) isolates, and the cabinets (7/42 isolates, 16.6%), of which both were placed in the ICU and renal units **(Fig 4A).** Overall, coagulase-negative *Staphylococci* isolates (CONS) were the most common bacteria isolated from high-contact surfaces (38%, 16/42). This was followed by *S*. *aureus* (14.3%, 6/42) and *P*. *luteola* (9.5%, 4/42) (**Fig 4B**) all representing environmental swabs cultured from the renal unit and ICU. The environmental isolates from the ICU had *S*. *aureus* as the most common bacteria at 15.8% (6/38). In the renal unit, four isolates were evenly distributed in the environment, namely, *S*. *epidermidis*, *S*. *lentus*, *E*. *cloacae*, and *Y*. *pseudotuberculosis*.

### Antimicrobial susceptibility profiles of enteric bacteria and ESBL phenotype

**Fig 5:**
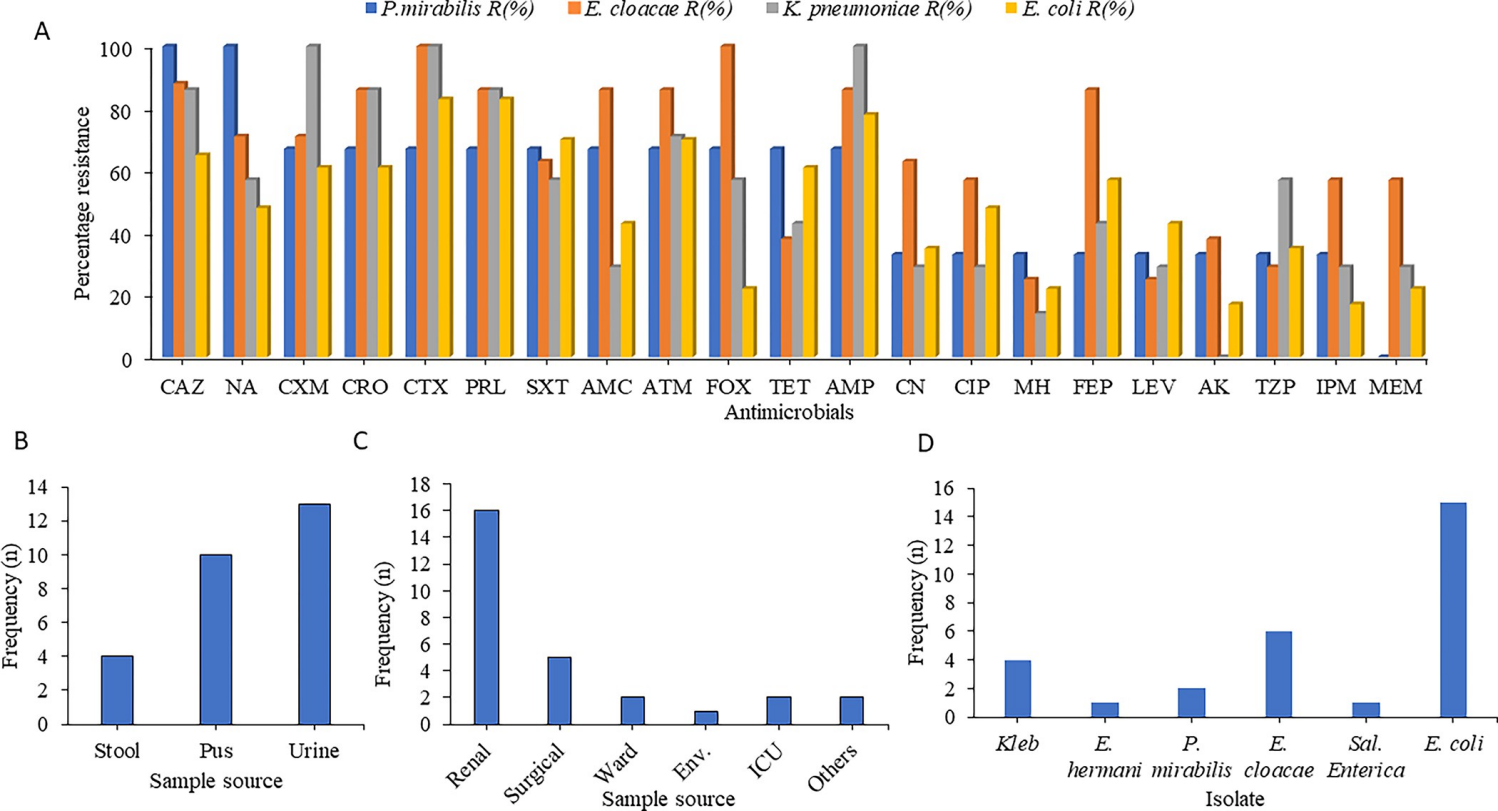
(A)Antibiotic resistance profiles of Enteric bacteria isolates (B) Distribution of ESBL phenotype isolates from different sections and wards (C)Distribution of samples from which bacteria portraying ESBL phenotype were isolated; (D) Frequency of enteric isolates with ESBL phenotype.

AMR phenotypes of interest yielded 29 isolates with ESBL phenotype, which we characterized in this paper. Additionally, we characterized *A*. *baumannii*, *S*. *maltophilia*, and *B*. *cepacia* resistance profiles. Overall, high resistance rates were observed against the third-generation cephalosporins, monobactams, nalidixic acid, tetracyclines, and penicillins. Between 75–87% of the microorganisms were resistant to cephalosporins and ampicillin. A moderate level of resistance was observed against fourth-generation cephalosporins (cefepime), amoxicillin clavulanate, piptazobactam, ciprofloxacin, sulphamethoxazole and gentamycin (50–65%) of the microorganisms. Low levels of resistance were observed against amikacin, minocycline, meropenem, and imipenem (22–45% of the microorganisms). It is worth noting that the *E*. *cloacae* isolates were exceptionally highly resistant to antibiotics that other enterics were moderately resistant against e.g. cefepime **(Fig 5A).**

Cefotaxime and piperacillin resistance among *E*. *coli* isolates was the highest at 83% (19/23). Ampicillin resistance rate came in second at 18/23 78%. Sulphamethoxazole rates of resistance followed at 70% (16/23). Ceftazidime (15/23) and aztreonam (15/23) resistance rates were both at 65.2%. Amikacin, cefoxitin, meropenem, and imipenem remained highly effective against the *E*. *coli* isolates with resistance rates of 17% (4/23), 22% (5/23), 22% (5/23), and 17% (4/23) respectively **(Fig 5A).** All the *K*. *pneumoniae* isolates in this study remained highly resistant against cefuroxime and cefotaxime at a rate of 100% (6/6). Ceftriaxone, piperacillin, and ceftazidime-resistant rates were also high at 83% (5/6). All *K*. *pneumoniae* isolates were susceptible to amikacin. Minocycline, ciprofloxacin, amoxicillin/clavulanate, imipenem, and meropenem were highly effective against the *K*. *pneumoniae* isolates with resistance levels of 17% (1/6), 33.2% (2/6), 33.2% (2/6) and 33.2% (2/6) respectively **(Fig 5A).** Statistical analysis showed that there was no significant difference in resistance rates across the different enteric bacteria p>0.05.

All the *Proteus mirabilis* isolates in this study remained highly resistant against ceftazidime (3/3) and nalidixic acid (3/3). Resistance against cefuroxime, ceftriaxone, cefotaxime, and piperacillin came in second amongst *P*. *mirabilis* isolates at a rate of 67% (2/3). None of the *P*. *mirabilis* isolates was resistant to meropenem (0/3) and the resistance rate to imipenem was at 33% (1/3) (**Fig 5A**). All the *Enterobacter* cloacae isolates in this study remained highly resistant against cefotaxime, and cefoxitin at a rate of 100% (8/8). Ceftriaxone, piperacillin, amoxicillin, and aztreonam were highly resistant as well at a rate of 87% (7/8). Levofloxacin and minocycline were the most effective drugs against *E*. *cloacae* with a resistance rate of 25% (2/8) (**Fig 5A**). The only *S*. *enterica* isolate from this study was resistant to all cephalosporins tested, monobactam, piperacillin, sulphamethoxazole, ampicillin, tazobactam-piperacillin, amoxicillin clavulanate, imipenem and meropenem.

A microorganism is presumed to be an ESBL producer when the diameters of zones of inhibition are below 25 mm for ceftriaxone, 27 mm for cefotaxime, 27mm for aztreonam, and 22mm for ceftazidime as per CLSI guidelines. A total of 29 out of 44 (65.9%) isolates met this criterion and were thus presumed to have the ESBL phenotype. In this study, renal unit isolates had the highest number of isolates with ESBL phenotype, followed by the surgical ward and the medical ward (**Fig 5B)**. Urine samples analysed had the highest number of bacteria with ESBL phenotype followed by pus and stool samples **(Fig 5C)**. The majority of *E*. *coli* isolates (15/44, 34%) exhibited the ESBL phenotype, followed by *E*. *cloacae* (6/44, 13.6%) and *K*. *pneumoniae* (4/44, 9.09%) isolates (**Fig 5D)**. The isolates were resistant to third-generation cephalosporins and aztreonam in addition to many other antimicrobial groups such as tetracyclines and fluoroquinolones (**Fig 5D** and **Table 3)**. Most of these isolates were susceptible to amikacin.

**Table 3:** Enteric bacteria and the antibiotics they were resistant against.

Isolate	Source of isolate	Number of isolates (N)	Antimicrobials ineffective to enteric bacteria
***K*. *pneumoniae***	Pus	3	CXM, CRO, CTX, PRL, SXT, AMC, ATM, CAZ, FOX, FEP, TZP, NA, AMP
***E*. *hermanii***	Stool	1	CXM, CRO, CTX, PRL, ATM, CAZ, CIP, FEP, LEV, TZP, NA, AMP
***P*. *mirabilis***	Urine	1	CXM, CRO, CTX, PRL, AMC, ATM, CAZ, FOX, IPM, AMP
***E*. *cloacae***	Pus	2	CXM, CRO, CTX, PRL, SXT, AMC, CN, CAZ, FOX, TET, NA, AMP
	Urine	5	CXM, CRO, CTX, PRL, SXT, AMC, CN, ATM, CAZ, CIP, FOX, FEP, NA, AMP
***E*. *coli***	Pus	1	CXM, CRO, CTX, PRL, SXT, AMC, CN, ATM, CAZ, CIP, FOX, FEP, LEV, TET, NA, AMP
	Urine	7	CXM, CRO, CTX, PRL, ATM, CAZ, CIP, FEP, LEV, NA, AMP
	Stool	3	CXM, CRO, CTX, PRL, SXT, AMC, CN, CAZ, CIP, FEP, MH, LEV, TZP, TET, NA, AMP
***S*. *enterica***	Pus	3	CXM, CRO, CTX, PRL, SXT, AMC, ATM, CAZ, CIP, FOX, FEP, LEV, TZP, TET, NA, AMP
	Pus	1	CXM, CRO, CTX, PRL, SXT, AMC, ATM, CAZ, FOX, FEP, TZP, AMP
Total		27	

CXM = Cefuroxime, CRO = Ceftriaxone, CTX = Cefotaxime, PRL = Piperacillin, SXT = Sulphamethoxazole, AMC = Amoxicillin/ Clavulanate, CN = Gentamycin, ATM = Aztreonam, CAZ = Ceftazidime, CIP = Ciprofloxacin, FOX = Cefoxitin, FEP = Cefepime, LEV = Levofloxacin, TET = Tetracycline, NA = Nalidixic acid, AMP = Ampicillin, TZP = Piptazobactam

#### Antimicrobial susceptibility profiles of *Acinetobacter spp*, *B*. *cepacia*, and *S*. *maltophilia*

**Fig 6:**
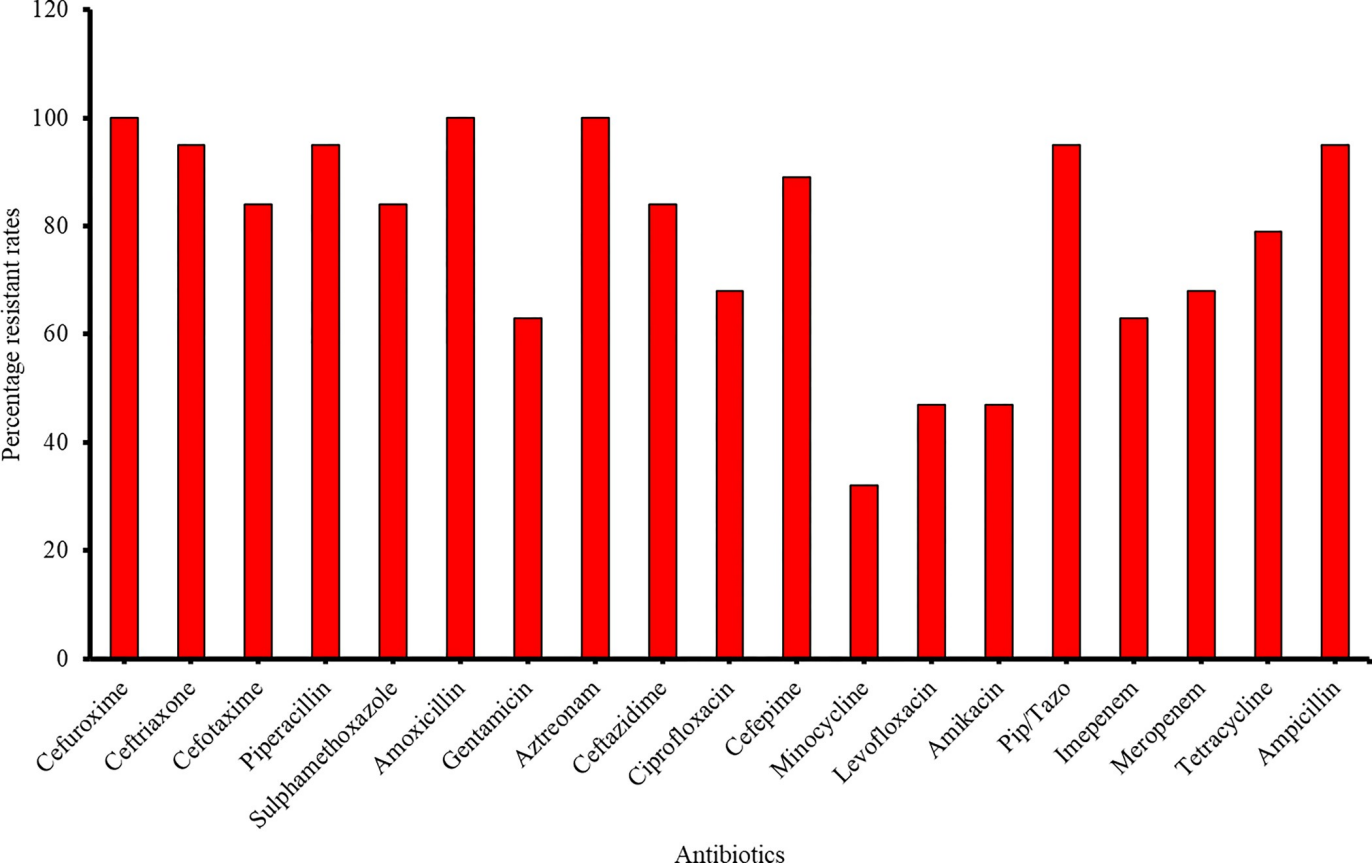
Resistance profiles of *A*. *baumannii*.

*A*. *baumannii* isolates were highly resistant to almost all groups of antibiotics including third and fourth-generation cephalosporins, monobactams, aminoglycosides, and the penicillin. For example, resistance to cefuroxime and aztreonam was at a rate of 100% (**Fig 6)**. Considerable resistance to carbapenems was observed for these isolates. A high resistance rate to β-lactam inhibitors was observed with 100% for amoxicillin clavulanate and 95% for piptazobactam. Low levels of resistance were however recorded against minocycline 32%, levofloxacin (47%), and amikacin (47%).

*B*. *cepacia* and *S*. *maltophilia* isolates were resistant to almost all groups of antibiotics tested. Based on the Clinical Laboratory Standard Institute Guidelines (2020) [20], interpretive zones for ceftazidime, meropenem, minocycline, and sulphamethoxazole, all our *B*. *cepacia* isolates were found to be resistant against these four antibiotics. The *S*. *maltophilia* isolate was resistant to SXT and minocycline but was susceptible to levofloxacin **(Table 4)**.

**Table 4:** Antibiotic resistance results for *B*. *cepacia* and *S*. *maltophilia*.

Bacteria	Antibiotic agent	Result	Number
*B*. *cepacia*	Ceftazidime	Resistant	2/2
	Meropenem	Resistant	2/2
	Minocycline	Resistant	2/2
	Sulphamethoxazole	Resistant	2/2
*S*. *maltophilia*	Sulphamethoxazole	Resistant	1/1
	Levofloxacin	Sensitive	1/1
	Minocycline	Intermediate	1/1

### Antimicrobial resistance profiles *for S*. *aureus* and CONS

*Staphylococci spp* recovered from this study demonstrated a high level of resistance to penicillin (97%), cefoxitin 19/32, 59% for *S*. *aureus* and 28/37, 76% for CONS. Resistance to amoxicillin-clavulanate (20/32, 63%) for *S*. *aureus* and 24/37, 68% for CONS. The most effective antimicrobials against the *Staphylococci* were gentamycin and levofloxacin, both with a 6/32, 19% (6/32) resistance rate for *S*. *aureus*. Susceptibility of CONS to gentamycin and levofloxacin was 41% and 54% to levofloxacin respectively. Of the *Staphylococci* isolates, 68% (47/69) were resistant to cefoxitin (a surrogate for methicillin resistance) and thus selected for future genotypic analysis for mecA gene detection **(Table 5)**.

**Table 5:** Antimicrobial resistance patterns of *Staphylococci spp*.

	*S*. *aureus*			Coagulase-negative *Staphylococci*		
Antibiotic	R nr/n (%)	I ni/n (%)	S ns/n (%)	R nr/n (%)	I ni/n (%)	S ns/n (%)
Erythromycin	(15/32) 47.00	(12/32) 38.00	(5/32) 16.00	(25/37) 68	(11/37) 30	(1/37) 3
Amoxicillin/Clavulanate	(20/32) 63.00	(1/32) 3.00	(11/32) 34.00	(25/37) 68	(3/37) 8	(9/37) 24
Sulfamethoxazole/Trimethoprim	(10/32) 32.00	(2/32) 6.00	(19/32) 97.00	(24/37) 65	(1/37) 3	(12/37) 32
Penicillin	(31/32) 97.00	(0/32) 0.00	(1/32) 3.00	(36/37) 97	(0/37) 0	(1/37) 3
Ceftriaxone	(15/32) 47.00	(9/32) 28.00	(8/32) 25.00	(26/37) 70	(7/37) 19	(4/37) 11
Tetracycline	(14/32) 44.00	(6/32) 19.00	(12/32) 38.00	(21/37) 57	(3/37) 8	(13/37) 35
Gentamycin	(6/32) 19.00	(1/32) 3.00	(25/32) 78.00	(15/37) 41	(3/37) 8	(9/37) 51
Oxacillin	(27/32) 84.00	(0/32) 0.00	(5/32) 16.00	(37/37) 100	(0/37) 0	(0/37) 0
Clindamycin	(16/32) 50.00	(5/32) 16.00	(11/32) 34.00	(23/37) 62	(9/37) 24	(5/37) 14
Levofloxacin	(6/32) 19.00	(2/32) 6.00	(24/32) 75.00	(20/37) 54	(1/37) 3	(16/37) 43
Cefoxitin	(19/32) 59.00	(0/32) 0.00	(13/32) 41.00	(28/37) 76	(0/37) 0	(9/37) 24
Cloxacillin	(22/32) 69.00	(2/32) 6.00	(8/32) 25.00	(32/37) 86	(0/37) 0	(5/37) 14

nr = number of resistant isolates, ni = number of intermediate resistant isolates, ns = number of susceptible isolates

## Discussion

Bacterial infections contribute to patients’ poor prognosis and increased risk of ICU admission and mortality [4]. The present study reveals a high culture positivity which was higher than that reported in Rwanda [19] and South Ethiopia [21]. Among the positive samples, those associated with females were less than those from males contrary to other studies which demonstrated more samples from females than males. This was mainly due to the sharp increase in motorcycle accidents in Kenya, which mainly affects males [22]. Gram-negative bacteria were the more dominant isolates similar to other studies conducted in Africa [21, 23]. Overall, coagulase-negative *Staphylococci* were the most isolated bacterial isolates, comparable to other studies that indicate CONS as the most commonly isolated microorganisms in clinical settings [24].

The bacterial spectrum was typical to other studies in Eastern Africa [25–29] but here we describe the *S*. *maltophilia* and *B*. *cepacia* for the first time in our study site from pus and urine samples in both medical and surgical wards. *S*. *maltophilia* is a multidrug Gram-negative bacillus that is an opportunistic pathogen, particularly for hospitalized patients [30] while *B*. *cepacia* is resistant to multiple antimicrobials and its treatment poses a challenge [31].

In the present study, most of the positive samples from the environment were from the ICU and constituted *S*. *aureus*. Similar results have been reported in Ethiopia [32]. The low number of positives in the renal unit may be attributable to frequent fumigation that is done at the renal unit, specifically after the dialysis sessions and effective infection prevention control mechanisms. These findings are echoed by other studies, which have reported no growth from dialysis ward bacterial surface culture in Northwest Ethiopia [33].

Among the different surfaces and inanimate objects examined, the highest bacterial contaminated samples were taken from bed rails, cabinets, and monitors, corroborating the findings from similar studies in Iran and Nigeria that identified beds as highly contaminated [34–36]. Overall, CONS were the most frequently isolated bacteria followed by *S*. *aureus*, consistent with findings from different studies from Ethiopia and elsewhere [35, 37]. As for *P*. *luteola*, it made up 9.5% of the isolates. The rate of isolation is comparable to other similar studies [38]. Trauma patient samples yielded the largest number of bacterial isolates (33.1%) since most of them had undergone orthopaedics surgery and were in the surgical ward. This finding is similar to a previous study that recorded high rates of bacterial infection in orthopaedics patients at a Tertiary Care Hospital in Bengaluru [39].

In this study, *A*. *baumannii* was highest in number amongst isolates from the surgical ward. Other studies have demonstrated that this species is frequently associated with skin and tissue infections at surgical sites [40, 41]. Infections due to *S*. *aureus* were also high among all medical ward isolates.

In the present study, the sampling points for urine samples were ICU, surgical, and medical wards, which have long-term admissions and catheterization. It has been reported that catheterization increases the incidence of urinary tract infection (UTI) by 3–5% and long admission increases the probability of acquiring UTI since hospitals are one of the sources of infection [42]. Renal unit patients were revealed to be associated with the highest positive bacterial cultures of urine samples (50%) compared to all other clinical samples. This finding confirms that individuals with dysfunctional kidneys are more prone to urinary tract infections as previously reported by a study in Saudi Arabia [37].

Resistance to antimicrobial agents is a problem in healthcare facilities, but in hospitals, transmission of bacteria is amplified because of the highly susceptible population [43]. The observed high resistance rates suggest poor adherence to antibiotic use guidelines and infection prevention policies in our study setting and beyond [44, 45]. The antibiotic sensitivity test results of our study confirmed that an alarming percentage of resistance was exhibited by bacterial isolates to the commonly prescribed antibiotics. Among the ESBL producers, the highest rates were observed amongst *E*. *coli* (32.6%) similar to other studies [46–48] and they were followed by *E*. *cloacae* isolates (18.6%). It has been reported that *E*. *cloacae* have a selective advantage over other bacteria to produce Amp C when there is antibiotic pressure [49]. The *E*. *cloacae* in this study demonstrated 100% resistance to cefotaxime and cefoxitin. Resistance to cephalosporins is mostly pronounced in these species [42]. Enterobacteria from this study remained highly resistant to cefuroxime, cefotaxime, piperacillin, and cefoxitin in general similar to a previous study in Dakar [50]. On the other hand, meropenem, imipenem, levofloxacin, and minocycline remained highly effective amongst the enteric bacteria. ESBL prevalence of 65.9% in our study is comparable to a similar study in Kenya amongst severely ill COVID-19 patients of 67.3% [51]. However, this rate is higher than a rate reported in Tanzania (33.14%) [52] and lower than that of Uganda (89%) [53]. African countries with high rates of ESBL producers according to Onduru *et al*. were Democratic Republic of Congo (92%), Tanzania (89%) and Malawi at 62% [54].

*K*. *pneumoniae* isolates in this study were also highly resistant to cephalosporins but susceptible to minocycline, meropenem, and imipenem. This pattern was also noted for *P*. *mirabilis* isolates. *E*. *coli* isolates highest resistance rates were against cefotaxime and piperacillin. Resistance rates for sulphamethoxazole were at 71% while that of ceftazidime and aztreonam were at 67%. The effective drugs against *E*. *coli* were amikacin and carbapenems. This is consistent with a similar study carried out elsewhere (39). *A*. *baumannii*, *B*. *cepacia*, and *S*. *maltophilia* isolates were highly resistant to all groups of drugs and this is consistent with findings from other studies in Kenya and beyond [55, 56].

The overall prevalence of MRSA was 59% (19/32). Recent studies indicate that disc diffusion test using cefoxitin is far superior to most of the currently recommended phenotypic methods like oxacillin disc diffusion. It has been reported as surrogate marker of *mecA* gene, gives clearer end points, easier to read and is more reproducible than tests with oxacillin disk diffusion. Thus, cefoxitin is now an accepted method for detecting MRSA with high efficiency and has been used as an alternative to PCR in resource constrained areas. This MRSA prevalence is higher compared to 22.6% from a similar study in Nigeria [57]. In Kenya, the prevalence of MRSA from previous studies was found to range from 1–84% [58]. One key limitation of this study is the absence of anaerobic cultures to identify anaerobic bacteria responsible for hospital-associated infections. Thus, it is important to highlight the need for future research to include anaerobic culture methods to adequately identify and understand the impact of these bacteria in hospital settings.

## Conclusions

This study recorded the presence of multidrug-resistant organisms of concern including recently emerged ones such as *S*. *maltophilia* and *B*. *cepacia* at Thika Level V Hospital. The implications of these organisms on patient outcomes and the potential for outbreaks are significant, necessitating the adoption of robust strategies to limit their spread. Encouraging judicious use of antimicrobial agents, implementing effective waste-handling protocols, and regular cleaning of high-touch surfaces, are critical steps towards reducing bacterial loads and preventing hospital-acquired infections. In addition, periodic monitoring systems should be implemented to enable prompt detection and management of infections. This study underscores the urgent need for continued efforts to mitigate the emergence and transmission of multidrug-resistant organisms in hospitals.

## Supporting information

S1 File (XLSX)

S2 File (XLSX)
